# A Comparative Assessment of Biological Effects and Chemical Profile of Italian *Asphodeline lutea* Extracts

**DOI:** 10.3390/molecules23020461

**Published:** 2018-02-19

**Authors:** Dora Melucci, Marcello Locatelli, Clinio Locatelli, Alessandro Zappi, Francesco De Laurentiis, Simone Carradori, Cristina Campestre, Lidia Leporini, Gokhan Zengin, Carene Marie Nancy Picot, Luigi Menghini, Mohamad Fawzi Mahomoodally

**Affiliations:** 1 Department of Chemistry “G. Ciamician”, University of Bologna, 40126 Bologna, Italy; dora.melucci@unibo.it (D.M.); clinio.locatelli@unibo.it (C.L.); alessandro.zappi4@unibo.it (A.Z.); francesco.de5@studio.unibo.it (F.D.L.); 2 Department of Pharmacy, University “G. D’Annunzio” of Chieti-Pescara, 66100 Chieti, Italy; simone.carradori@unich.it (S.C.); cristina.campestre@unich.it (C.C.); l.leporini@unich.it (L.L.); luigi.menghini@unich.it (L.M.); 3 Interuniversity Consortium of Structural and Systems Biology, 00136 Rome, Italy; 4 Department of Biology, Selcuk University, Campus, 42250, 42130 Konya, Turkey; gokhanzengin@selcuk.edu.tr; 5 Department of Health Sciences, University of Mauritius, Réduit 80837, Mauritius; picotcarene@yahoo.com (C.M.N.P.); f.mahomoodally@uom.ac.mu (M.F.M.)

**Keywords:** *Asphodeline lutea*, HPLC-PDA, heavy metals, tyrosinase, diabetes, neurodegenerative disease

## Abstract

The present study aims to highlight the therapeutic potential of *Asphodeline lutea* (*AL*), a wild edible plant of the Mediterranean diet. Roots, aerial parts, and flowers of *AL* at two different phenological stages were collected from three locations in Italy. The inhibitory activities of extracts on strategic enzymes linked to human diseases were assessed. The antioxidant properties were evaluated in vitro, using six standard bioassays. The phenolic and anthraquinone profiles were also established using HPLC-PDA. Zinc, cadmium, lead, and copper contents were also determined. All the samples inhibited acetylcholinesterase (from 1.51 to 2.20 mg GALAEs/g extract), tyrosinase (from 7.50 to 25.3 mg KAEs/g extract), and α-amylase (from 0.37 to 0.51 mmol ACAEs/g extract). Aloe-emodin and physcion were present in all parts, while rhein was not detected. The phenolic profile and the heavy metals composition of specimens gathered from three different regions of Italy were different. It can be argued that samples collected near the street can contain higher concentrations of heavy metals. The experimental data confirm that the *A. lutea* species could be considered as a potential source of bioactive metabolites, and its consumption could play a positive and safe role in human health maintenance.

## 1. Introduction

*Asphodeline lutea* (*AL*) Reichenb (synonym: *Asphodelus luteus* L., family Xanthorrhoeaceae), also known as King’s Spear or Yellow Asphodel, is a perennial landscaping plant native to South-eastern Europe, North Africa and Turkey, characterized by a single stem with semi hollow leaves and yellow-orange flowers [1,2]. The stems and leaves are traditionally consumed in the Mediterranean region as an edible plant due to their nutritional protein quality of [3,4].

The chemotherapeutic value of Bulgarian and Turkish *AL* root extracts has been evidenced only in recent years, revealing the presence of different therapeutically useful compounds. The anti-microbial and anti-mutagenic activities of methanol root extracts of *AL* have been reported [5], while the hepatoprotective and antioxidant capacity of the ethanol root extracts of *AL* both in vivo and in vitro has also been evidenced in an animal model of CCl_4_-injured liver [6]. Moreover, the methanol and chloroform extracts from *AL* roots caused a marked inhibition of multidrug resistance in mouse tumour cells transfected with the human *MDR1* gene [7], whereas methanol, acetone and aqueous extracts of different *Asphodeline* spp. parts were reported to moderately inhibit elastase, collagenase and hyaluronidase enzymes at 100 µg/mL [8]. Other studies have reported the use of extracts of *A. lutea* among local populations for skin diseases and haemorrhoids [9,10].

The methanol root extracts of *AL* of Bulgarian origin were found to be rich in caffeic acid, catechin and epicatechin [11]. Anthraquinones (1,5,8-trihydroxy-3-methylanthraquinone, 1-hydroxy-8-methoxy-3-methylanthraquinone, chrysophanol, 1,1′,8,8′,10-pentahydroxy-3,3′-dimethyl-10,7′-bianthracene-9,9′,10′-trione) [12], naphthalenes and naphthoquinones [13] were also previously isolated from *AL*. The antioxidant activity of *AL* chloroform extracts in lard and sunflower oil was attributed to 2-acetyl-1-hydroxy-8-methoxy-3-methylnaphthalene [14]. However, little is known about the chemical variability and the potential therapeutic ability of *AL* of Italian origin.

Based on these considerations, we aimed to evaluate the antioxidant activities, the enzyme (acetylcholinesterase, butyrylcholinesterase, tyrosinase, α-amylase, and α-glucosidase) inhibitory potential of extracts from different anatomical regions of *AL,* collected in diverse sites in the Italian Central Apennines, at different phenological stages, as well as the determination of anthraquinones, phenolics and heavy metal profiles.

## 2. Results

After extraction, each sample was fully characterized to establish a comprehensive chemical fingerprint of total phenolic and flavonoid content (Table 1), specific content of phenolics (Table 2), and anthraquinones (Table 3) and heavy metals bioaccumulation (Table 4). Then, the *AL* extracts were tested in order to assess their pharmacological properties such as antioxidant, metal chelating and enzyme inhibition.

From the data in Table 1 it is possible to state that both phenolic and flavonoid contents reached the maximum in the flowering stage with respect to the preflowering one. Aerial parts were the richest in these metabolites (AP ˃ Fl ˃ R) disregarding the sampling site. For the phenolic content, the following order was observed: aerial parts of *AL* collected in Pescosansonesco > Perugia > Novele. A similar pattern was observed for the flavonoid content. The lowest amount of phenolics was recorded in the roots of the preflowering plant. The phenolic content in whole plant increases evidently during blooming, and this aspect is mainly related to the flowers development, while the amount of phenolics in roots and aerial parts were constant. The contribution of the flavonoid fractions, which represent more than fifty percent of the floral phenolic content, is particularly evident. The distribution of phenolics mainly in the aerial parts is consistent with the physiological function of such class of metabolites, and could support their nutraceutical value when used as edible parts. These data showed some differences with total phenolic content (13.02 mg GAE/g DW) and total flavonoid content (7.63 mg RE/g DW) found in the roots of *A. lutea* from Syrian origin [15]. 

The detailed phenolic profiles of the tested extracts of *AL* are summarized in Table 2. The aerial part of preflowering *AL* (86.15 µg/mg) collected in Pescosansonesco contained the highest amount of phenolic compounds, with a high quantity of 2,3-dimethoxybenzoic acid. Benzoic acid and quercetin were present in the aerial parts of flowering *AL* collected in all the three locations. Aerial parts of preflowering *AL* (0.588 µg/mg) collected in Novele contained the lowest amount of phenolic compounds (rutin 0.28 ± 0.04 µg/mg and quercetin 0.31 ± 0.05 µg/mg). Chlorogenic acid, vanillic acid, syringic acid, 3-hydroxy-4-methoxybenzaldehyde, sinapic acid, *trans*-ferulic acid, *o*-coumaric acid, and *trans*-cinnamic acid were not detected in any sample. Naringenin was found only in aerial parts and only in two samples (Fl from Perugia and AP from Novele at the preflowering stage). On the other hand, epicatechin was detected only in roots from plants collected in Perugia in full bloom. Naringin, naringenin, benzoic acid and its derivatives (2,3-dimethoxybenzoic acid, 3-hydroxybenzoic acid, 4-hydroxybenzoic acid), gallic acid, catechin and epicatechin, *p*-coumaric acid and rutin were identified and quantified in this species for the first time. This information could enhance the knowledge about the nutritional and medical value of this plant.

The quantification of anthraquinones in the extracts of *AL* collected from different spontaneous growing sites in Italy is presented in Table 3. Aloe-emodin and physcion were present in all the extracts, whereas rhein was not detected. Emodin was present only in the roots collected before flowering in the samples from Pescosansonesco. The roots proved to be the plant part with the highest anthraquinone content. This quantity was higher in samples of non-flowering plants. The data are consistent with the general rules indicating the preferred harvest time for hypogeous organs is during the vegetative stage.

Table 4 reports the extrapolated concentrations of Zn(II), Cd(II), Pb(II), and Cu(II) in all the extracts. The *AL* samples collected in Pescosansonesco showed contents of all metals lower than the limit of detection (LoD) or too low to be detectable by the polarograph (whose LoD is usually on the order of 10 ppb). The Perugia samples showed the presence of Zn(II) in almost all parts of the plant, both before and after flowering, and a low amount of Cd(II) and Cu(II) in post-flowering parts of the plant. The samples collected in Novele, instead, showed a significant content of Zn(II), Cd(II) and Cu(II), mostly in post-flowering aerial parts and flowers. 

This higher concentration of metals in the plants of Novele can be explained by the fact that their sampling sites were closer to vehicular traffic. Thus, it is conceivable that the *AL* collected in the proximity of main roads can contain high amounts of heavy metals due to car traffic and anthropogenic factors, but it could be important to consider the possibility of potential correlation of pollutants, as external stressors, that can act as modulator of plant secondary metabolism.

Table 5 summarizes the radical scavenging, reducing, antioxidant, and metal chelating properties of the plants parts of *AL* collected in Perugia, Novele, and Pescosansonesco. The following order was observed for DPPH radical scavenging of samples collected from the three locations: aerial parts of flowering *A. lutea* > aerial parts of preflowering *A. lutea* >flowers of flowering *A. lutea* > roots of flowering *A. lutea* > roots of preflowering *A. lutea*. Similar promising results were also reported by other authors regarding *A. lutea* species from different origin [15,16]. The aerial parts of flowering *A. lutea* collected from Perugia and Pescosansonesco showed potent reducing capacity against FRAP (84 and 128 mg TEs/g extract, respectively) and CUPRAC (108 and 160 mg TEs/g extract, respectively). The roots of flowering *A. lutea* collected in Perugia showed the highest antioxidant activity. Conversely, aerial parts of *A. lutea* harvested in Novele showed the most potent metal chelation ability (Table 5).

Table 6 presents the inhibitory activity of extracts from different parts of the plant (aerial part, flowers, and roots) of *AL* collected from three separate accessions from Central Italy. All the studied samples inhibited AChE, with values ranging from 1.51 to 2.20 mg GALAEs/g extract. Conversely, other authors found a very low AChE inhibition for both leaves and bulb extracts of *A. lutea* from Palestinian flora [16]. Similarly, the studied plant samples inhibited tyrosinase (ranging from 7.50 to 25.30 mg KAEs/g extract) and α-amylase (ranging from 0.37 to 0.51 mmol ACAEs/g extract). The aerial parts of preflowering *AL* collected in Perugia and those of flowering *AL* collected in Pescosansonesco showed no activity against BChE. The best α-glucosidase inhibitory effects (44.2 mmol ACAEs/g extract) was observed in the roots of flowering *AL* collected in Novele.

## 3. Discussion

Chronic pathologies, such as type II diabetes and neurodegenerative diseases such as Alzheimer’s disease (AD) are pandemics affecting every segment of the population. Existing therapies alleviate the trauma caused by these pathologies, but harbour several side effects. The concern of the scientific community is to find novel therapeutic agents with a minimum of side effects, to manage type II diabetes and AD. Plants possess a large array of phenolic compounds endowed with numerous therapeutic properties.

Neurodegenerative disorders encompass more than 600 diseases involving progressive and irreversible deterioration of the nervous system, subsequently resulting in neuronal cell death [17]. The most prevalent form of neurodegenerative disorder afflicting the global population is AD. AD is believed to occur because of the accumulation of amyloid-beta protein and the alteration of tau proteins in the brain, and an apparent oxidative stress [18]. A hypothesis has been suggested that AD is related to type II diabetes, though the association is complex and not fully understood. Notwithstanding, conditions are interlinked by inflammatory response, insulin resistance, glycogen synthase kinase 3β signalling mechanism, insulin growth factor signalling, oxidative stress, neurofibrillary tangle formation, acetylcholinesterase activity regulation, and amyloid beta formation. Thus, the inhibition of acetylcholinesterase, butyrylcholinesterase, tyrosinase, α-amylase, and α-glucosidase has been considered to mitigate the deleterious effects of type II diabetes and AD or as adjunctive treatment modalities [19]. Additionally, scientific interest is gradually shifting towards enzymes which have not yet been considered by the pharmaceutical industries. The inhibition of such enzymes, so far considered as non-pharma target, can be of potential relevance and can proved to be a promising strategy for the management of these debilitating complications [20].

AChE has attracted much attention for the management of this devastating irreversible disorder, owing to its ability to hydrolyse acetylcholine, a neurotransmitter [21]. The activity of BChE was found to increase in areas of the brain most affected by AD [22]. Furthermore, in the late stage of Alzheimer’s disease, the level of AChE was found to drop to 55–67% of normal value, while BChE activity increases up to 120% [23]. Finding cholinesterase inhibitors having different specificities might help to allay the trauma associated to the different stages of AD.

In the present study, the aerials parts of flowering *AL* collected from Pescosansonesco showed potent inhibition of AChE, but no activity against BChE. Wang and co-workers previously reported that aloe-emodin was a potent inhibitor of AChE [24]. Interestingly, aerials parts of flowering *AL* from Pescosansonesco contained significant amount of aloe-emodin and physcion. *A. anatolica,* another species of the genus *Asphodeline*, was reported to contained physcion and also inhibited AChE [19]. The roots of *AL* at the preflowering stage collected at Perugia and Novele demonstrated potent BChE inhibitory activity. These plant samples contained high level of physcion. This anthraquinone was also previously reported to exert moderate to strong inhibition on tyrosinase [25]. Tyrosinase is a multifunctional copper-containing metalloenzyme required for the production of melanin pigment in humans [26]. Melanin plays a vital role by shielding ionizing radiation and absorbing free radicals [27]. Additionally, depletion of neuromelanin, which is structurally related to melanin, present in the substantia nigra, was associated to Parkinson’s disease [28]. The inhibition of tyrosinase might prevent the aggravation of neurodegenerative disorders, such as Parkinson’s disease. It was observed that mostly the aerial parts of *AL* possessed pronounced tyrosinase inhibitory potential. The aerial parts contained the highest amount of phenolic compounds. *AL* was used traditionally for the management of skin ailments. This virtue might be ascribed to its tyrosinase inhibitory potential, as reported in the present study.

Chrysophanol was identified in all the investigated samples. This anthraquinone was shown to inhibit mammalian intestinal α-glucosidase activity [29], thus delaying glucose surge. Most of the studied plant samples were potent inhibitors of α-glucosidase, showing higher acarbose equivalent values, compared to α-amylase inhibition. Indeed, mild α-amylase inhibition versus marked α-glucosidase inhibition was requested, since a pronounced activity of the former enzyme advocated the event of gastrointestinal problems [30]. Specifically, the roots of flowering *AL* collected in Novele showed high α-glucosidase inhibitory activity and low inhibition against α-amylase. This sample contained the highest amount of gallic acid, previously reported to possess α-amylase and α-glucosidase inhibitory activity [31], while *p*-coumaric acid was shown to be inactive [32]. Among the detected antraquinones, aloe-emodin, crysophanol and physcion can contribute to α-glucosidase inhibition [29,33]. The antioxidant potential of different parts of *AL* collected from different locations was also evaluated. Indeed, oxidative stress has been associated to the onset and/or worsening of various pathologies, including type II diabetes and AD. FRAP, CUPRAC and phosphomolybdenum assay are methods suitable to measure the reducing abilities of the samples. FRAP is based on the reduction of Fe^3+^–TPTZ (2,4,6-tri(2-pyridyl)-*s*-triazine) complex, to produce coloured Fe^2+^–TPTZ [24], Cu(II)–Nc (neocuproine) is reduced to coloured Cu(I)–Nc in the CUPRAC assay, while the phosphomolybdenum assay is based on the reduction of molybdenum (VI) to molybdenum (V) producing a green phosphomolybdenum (V) complex [34]. Aerial parts of *AL* collected in Pescosansonesco at flowering stage showed the highest reducing potential against the three reducing methods used. Indeed, this sample contained the highest amount of phenolics and flavonoids, known to possess strong reducing potential.

From the reported results is important to highlight that the activities obtained from phosphomolybdenum assay for the Italian *AL* roots (from 0.71 to 1.03 mmol TE/g extract) were lower than the corresponding *Asphodeline* spp. from Turkey [35,36,37] (from 1.18 to 2.94 mmol TE/g extract). No differences were observed for metal chelating activity assay.

Our CUPRAC and FRAP results are in accordance with the data reported in literature [35,36,37], even if is important to highlight that slightly lower values were observed for the Novele PF-R root samples. On the other hand, ABTS and DPPH assays measure the ability of the plant samples to scavenge free radicals. DPPH is a protonated free radical, which is reduced to a stable diamagnetic molecule [38]. The data collected in the present investigation demonstrated that aerial parts of flowering *AL* were the most potent scavengers of DPPH radical compared to the other extracts, irrespective of the sampling site. The herein reported DPPH and ABTS results are in accordance with the data reported in literature for root samples [35,36,37], even if the sample Perugia F-R shows on DPPH test the higher value (43.8 mg TE/g extract) respect to the other *Asphodeline* spp.

Additional comparisons could be done considering another edible plant, as just reported in literature [37,39]. Also in this case for *Asphodeline* root samples very similar biological activities respect to phosphomolybdenum, CUPRAC, FRAP, DPPH and ABTS assays were observed, and it is further confirmed that *Asphodeline* spp. had lower activity compared to *Potentilla* spp. [39,40,41].

The increased interest in the use of ABTS radical arises from its ability to act in both organic and aqueous conditions and its stability in a wide range of pH [42]. The aerial parts of flowering *AL* collected in Perugia and Pescosansonesco showed strong ABTS radical quenching abilities compared to the other plant parts as also reported by Karandeniz et al. [43]. Interestingly, the phenolic and flavonoid contents were the highest in the same samples. Previous studies have appraised the potent scavenging capacities of phenolics and flavonoids on ABTS [44]. 

Transition metals, especially Cu and Fe, act as catalysts in the production of reactive oxygen species, which react with other molecules resulting in oxidative stress and cell damage/death [45]. In vitro techniques used to assess the ability of phytochemicals to chelate these potentially toxic transition metals offer the scope for the development of nutraceuticals. In the present study, the different plants parts of *AL* showed variable degree of metal chelating potential. The preflowering aerial parts of *AL* collected in Novele showed the highest metal chelating ability. This sample contained rutin and quercetin, previously reported to exert metal chelating properties [46,47]. Comparing the chemical composition and activities of different parts of the same plant, as well as the parts from plants in different phenological stages, an evident quantitative and qualitative variability was recorded. Ecological, climatic, and genetic factors are probably involved in the variation of secondary metabolite profiles observed from samples of *AL* of different geographical origin [48,49].

## 4. Conclusions

The present study could be considered as the first comparative investigation on chemical composition (phenolics, anthraquinones and heavy metals) and biological activities of extracts from different parts of *A. lutea* collected from three different wild populations in Central Italy. Owing to the key role of oxidative stress and heavy metals in the onset/progression of large number of diseases, the antioxidant and chelating potential of the plant was also assessed. The phenolic and anthraquinone profile of *A. lutea* extracts evidenced qualitative and quantitative differences that, at least in part, are coherent with the presence of anthraquinones mainly in roots and phenolics in the aerial parts. The dynamics of production and accumulation of selected active metabolites during the floral induction phase are still not clear, apart from the quantity of phenolics in the aerial part that resulted strongly increased. Similarly, their enzyme inhibitory and antioxidant potential also varied. However, the potent enzyme inhibitory activity and antioxidant properties observed are worth further scientific consideration. The experimental data confirm that the *A. lutea* species could be rationally considered as a potential source of bioactive metabolites, and its consumption could play a positive role in human health maintenance.

## 5. Materials and Methods

### 5.1. Plant Materials

Plant material was collected from wild populations in three different locations in Central Italy, Perugia (43°06′39.2′′N 12°20′53.6′′E, 350 m a.s.l., in the surroundings of a residential building, probably subspontaneous), Novele (42°45′50.6′′N 13°20′35.0′′E, 550 m a.s.l., AP, close to the main road) and Pescosansonesco (42°14′31.4′′N 13°52′29.8′′E, PE, 530 m a.s.l., on a calcareous cliff). From each site a representative sampling (at least ten plants) was done, taking care to avoid causing damage to the wild population. Sampling was performed on March and May 2016, in order to collect plants during the vegetative phase (PF—pre-flowering stage) and in full bloom (F—flowering). The botanical identity was confirmed by a senior taxonomist (Prof. F. Tammaro) and voucher specimens are conserved in the Herbarium of the Department of Pharmacy, “G. d’Annunzio” University of Chieti-Pescara (Italy). Each plant was manually separated in root (R), aerial parts (AP, consisting in stem and leaves) and flowers (Fl) and air-dried in an oven at 40 ± 1 °C, until achievement of constant weight. Then plant material was ground using a mixer grinder to a fine powder, passing through a 40 mesh sieve to obtain a uniform granulometry and stored in a vacuum box in the dark at 4 °C until use. Methanol extracts were obtained by maceration with 250 mL of organic solvent at room temperature (25 ± 1 °C) overnight, as previously reported [50].

### 5.2. Chemicals

All the phenolic chemical standards (gallic acid, catechin, chlorogenic acid, *p*-hydroxybenzoic acid, vanillic acid, epicatechin, syringic acid, 3-hydroxybenzoic acid, 3-hydroxy-4-methoxy-benzaldehyde, *p*-coumaric acid, rutin, sinapic acid, *trans*-ferulic acid, naringin, 2,3-dimethoxy-benzoic acid, benzoic acid, *o*-coumaric acid, quercetin, *trans*-cinnamic acid, naringenin) (purity ˃ 98%) were purchased from Sigma Aldrich (Milan, Italy). Methanol (HPLC-grade), formic acid (99%), nitric acid (65%, supra-pure metal grade) and sulfuric acid (98%, ultrapure grade) were obtained from Carlo Erba Reagents (Milan, Italy). Double-distilled water was obtained using a Millipore Milli-Q Plus water treatment system (Millipore Bedford Corp., Bedford, MA, USA). Standard metal solutions of Cd(II), Cu(II), Pb(II) and Zn(II) (1000 mg L^−1^, suprapure grade), hydrochloric acid (30%) and sodium acetate (anhydrous, analytical grade) were purchased by Merck (Darmstad, Germany). Anthraquinone chemical standards (all ˃99%) were purchased from Extrasynthese (Genay, France).

### 5.3. Total Bioactive Components (Phenolics and Flavonoids)

Total phenolic content was determined using the Folin-Ciocalteu colorimetric method [51], and expressed as gallic acid equivalents (GAEs/g extract). Total flavonoid content was determined by previously reported method [52] and expressed as rutin equivalents (REs/g extract).

### 5.4. HPLC Analyses for Phenolics and Anthraquinones

High-performance liquid chromatography with diode array detection (HPLC-PDA) was employed. Phenolic as well as anthraquinone patterns were evaluated by validated methods reported in the literature [35,53], using an HPLC Waters liquid chromatography (model 600 solvent pump, 2996 DAD, Waters S.p.A., Milford, MA, USA). Mobile phase was directly degassed *on-line* using a Biotech 4CH DEGASI Compact (Onsala, Sweden). Empower v.2 Software (Waters S.p.A., Milford, MA, USA) was used to collect and analyse data.

For the quantitative analyses on investigated compounds (both anthraquinones and phenolics), the HPLC-PDA methods were validated using external calibration (for the identification of the analytes retention times, and UV/Vis spectra) with pure chemical standards at different concentration levels. Precision and trueness were validated using fortified samples (with pure chemical standard working solutions) at three different concentration levels and the results fulfil international guideline references [35,53]. The instrument configurations, using also a column oven for the reproducibility of the analytes retention times, and the validated methods allow to the correct identification and quantification of the investigated compounds. 

### 5.5. Heavy Metals Determination

Cd(II), Cu(II), Pb(II) and Zn(II) were determined by differential pulse adsorptive stripping voltammetry (DPASV). An AMEL Mod. 433 Multipolarograph (AMEL Instrumentation s.r.l., Milan, Italy), equipped with a hanging mercury drop electrode (HDME) as working electrode, Ag|AgCl|KCl_satd._ as reference electrode and a platinum-wire auxiliary electrode [54], was employed. The supporting electrolyte was an acetic acid/sodium acetate buffer (1 M, pH = 4.65).

The relevant instrumental parameters were the following. Initial potential: *E_i_* = −1.300 V; deposition potential: *E_d_* = −1.300 V; final potential: *E_f_* = 0.100 V; electrodeposition time: *t_d_* = 120 s; delay time before the potential sweep: *t_r_* = 5 s; potential scan rate: d*E*/d*t* = 20 mV/s; stirring rate: *r* = 600 r.p.m.; the potential values were referred to Ag|AgCl|KCl_satd._.

Each sample was previously digested by acid attack [55]: 0.25 g of plant sample was put in a 100-mL Pyrex digestion tube, and 20 mL of a mixture 1:1 of nitric acid and sulfuric acid was added. The tube, connected to a Vigreux column condenser, was then heated at 180 °C for 120 min. For the analytical measures, 15 mL of buffer solution and 300 μL of the digested solution were put into the voltammetric cell, and then 3 standard additions of 300 μL of a solution containing all the four metals at 10 mg L^−1^ were carried on. For each addition, two replicates were performed. The blank was obtained by mixing 15 mL of buffer solution and 300 μL of a digested solution containing only the mixture 1:1 of nitric acid and sulfuric acid. Finally, for each sample a standard addition line for each metal was computed, using peak areas as dependent variable and the metal content was extrapolated. The limit of detection (LoD) was calculated using the three-sigma approach, as in previous works [56].

### 5.6. Antioxidant Activity

The DPPH (1,1-diphenyl-2-picrylhydrazyl) radical and ABTS (2,2’-azino-bis(3-ethyl- benzothiazoline)-6-sulphonic acid) radical cation scavenging activities were determined, and the results were expressed as Trolox equivalents (TEs/g extract). The reducing power of the extracts was measured according to the reported method, using cupric ion reducing antioxidant power (CUPRAC) and ferric ion reducing antioxidant power (FRAP), and results were expressed as Trolox equivalents (TEs/g extract). Total antioxidant capacity was determined using phosphomolybdenum method. Metal chelating activity of the extracts against ferrous ions was also determined, and the results were expressed as EDTA equivalents (EDTAE/g extract). All these antioxidant procedures were performed as previously documented [57].

### 5.7. Enzyme Inhibitory Activities

Enzyme inhibitory activities (acetylcholinesterase (AChE), butyrylcholinesterase (BChE), tyrosinase, α-amylase and α-glucosidase) of the extracts were determined using the already published methodology [58]. Results for enzyme inhibitory activities were expressed as standard compound equivalents (galantamine for AChE and BChE, kojic acid for tyrosinase, acarbose for both α-amylase and α-glucosidase).

### 5.8. Statistical Analysis

All the assays were carried out in triplicate. The results are expressed as mean values and standard deviation (SD). The differences between some stages/parts in each location were calculated using one-way analysis of variance (ANOVA) followed by Tukey’s honest significant difference post hoc test with α = 0.05. This treatment was carried out using SPSS v. 14.0 program (SPSS Institute Inc., Cary, NC, USA).

## Figures and Tables

**Table 1:** Total phenolic and flavonoid content of different parts of *A. lutea* collected from three different locations in Italy.

Location	Stage/Parts	Total Phenolic Content (mg GAE/g Extract) *	Total Flavonoid Content (mg RE/g Extract) *
Perugia	PF-R	12.5 ± 0.2 ^a^	5.4 ± 0.7 ^a^
	PF-AP	24.7 ± 0.9 ^a^	19.8 ± 0.3 ^a^
	F-R	17.7 ± 0.4 ^a^	4.8 ± 0.1 ^a^
	F-AP	27.7 ± 0.6 ^b^	22.1 ± 0.2 ^b^
	F-Fl	19.4 ± 0.6 ^b^	11.4 ± 0.1 ^b^
Novele	PF-R	12.4 ± 0.7 ^a^	3.7 ± 0.2 ^b^
	PF-AP	23.8 ± 0.3 ^a^	14.8 ± 0.5 ^b^
	F-R	12.7 ± 0.2 ^b^	4.6 ± 0.1 ^a^
	F-AP	23.9 ± 0.3 ^c^	17.3 ± 0.1 ^c^
	F-Fl	17.5 ± 0.7 ^c^	11.0 ± 0.4 ^b^
Pescosansonesco	PF-R	9.8 ± 0.2 ^b^	2.9 ± 0.2 ^c^
	PF-AP	24.0 ± 0.8 ^a^	19.3 ± 0.2 ^a^
	F-R	10.7 ± 0.4 ^c^	3.2 ± 0.1 ^b^
	F-AP	38.2 ± 0.8 ^a^	28.0 ± 0.3 ^a^
	F-Fl	24.7 ± 0.5 ^a^	13.5 ± 0.3 ^a^

* Values expressed are means ± S.D. of three simultaneous measurements. GAEs, gallic acid equivalents; REs, rutin equivalents. PF: preflowering plant, F: flowering plant, R: roots, AP: aerial parts, Fl: flowers. Data marked with different letters within the same column indicate statistically significant differences in the same stages/parts for each location (*p* < 0.05).

**Table 2:** Phenolic profile of different parts of *A. lutea* collected from three different locations in Italy *.

Phenolic Components	Perugia					Novele					Pescosansonesco				
	PF-R	PF-AP	F-R	F-AP	F-Fl	PF-R	PF-AP	F-R	F-AP	F-Fl	PF-R	PF-AP	F-R	F-AP	F-Fl
Gallic acid	nd	nd	0.9 ± 0.1	nd	0.52 ± 0.05	nd	nd	1.1 ± 0.1	0.31 ± 0.05	1.1 ± 0.8	1.96 ± 0.04	nd	1.09 ± 0.02	1.1 ± 0.6	nd
Catechin	nd	0.84 ± 0.03	0.54 ± 0.05	nd	nd	0.57 ± 0.04	nd	nd	nd	nd	1.8 ± 0.1	1.1 ± 0.03	0.51 ± 0.01	nd	nd
*p*-OH benzoic acid	nd	nd	nd	nd	nd	nd	nd	nd	0.43 ± 0.07	nd	0.42 ± 0.05	nd	nd	nd	0.41 ± 0.02
Epicatechin	nd	nd	0.35 ± 0.03	nd	nd	nd	nd	nd	nd	nd	nd	nd	nd	nd	nd
3-OH benzoic acid	0.36 ± 0.04	nd	0.40 ± 0.05	nd	nd	nd	nd	nd	nd	nd	nd	1.9 ± 0.5	nd	nd	nd
*p*-Coumaric acid	nd	nd	nd	nd	nd	2.2 ± 0.2	nd	0.32 ± 0.05	nd	nd	0.24 ± 0.03	0.46 ± 0.03	nd	nd	0.42 ± 0.02
Rutin	nd	1.6 ± 0.9	nd	1.1 ± 0.1	9.0 ± 0.1	3.0 ± 0.3	0.28 ± 0.02	0.67 ± 0.08	nd	5.6 ± 0.4	10.0 ± 0.9	8.9 ± 0.7	0.31 ± 0.07	nd	0.9 ± 0.1
Naringin	nd	nd	nd	nd	nd	nd	nd	nd	nd	0.24 ± 0.03	nd	1.5 ± 0.2	0.28 ± 0.02	nd	nd
2,3-diMeOBA	nd	nd	0.33 ± 0.09	nd	nd	nd	nd	nd	0.37 ± 0.02	nd	nd	53.0 ± 5.0	nd	nd	nd
Benzoic acid	nd	1.2 ± 0.6	nd	0.50 ± 0.05	2.4 ± 0.2	0.56 ± 0.04	nd	0.26 ± 0.09	0.35 ± 0.01	nd	0.33 ± 0.02	18.0 ± 3.0	nd	0.4 ± 0.1	0.87 ± 0.09
Quercetin	0.31 ± 0.07	2.3 ± 0.3	nd	0.52 ± 0.05	2.3 ± 0.24	2.6 ± 0.2	0.31 ± 0.05	nd	2.7 ± 0.4	3.0 ± 1.0	12.0 ± 1.0	0.7 ± 0.1	0.63 ± 0.04	0.3 ± 0.8	5.0 ± 1.0
Naringenin	nd	nd	nd	nd	0.63 ± 0.03	nd	nd	nd	nd	nd	nd	0.79 ± 0.08	nd	nd	nd
Total (µg/mg)	0.67	6.01	2.54	2.12	14.88	8.94	0.59	2.31	4.20	10.29	27.32	86.15	2.81	1.89	7.66

* Values expressed are means ± S.D. of three simultaneous measurements. nd: not detected, PF: preflowering plant, F: flowering plant, R: roots, AP: aerial parts, Fl: flowers. 2,3-diMeOBA = 2,3-dimethoxy- benzoic acid. Chlorogenic acid, *trans*-cinnamic acid, *o*-coumaric acid, sinapinic acid, *trans*-ferulic acid, syringic acid, vanillic acid, and 3-OH-4-MeO-benzaldehyde were not detected in any sample.

**Table 3:** Anthraquinone profiles of different parts of *A. lutea* collected from three different locations in Italy *.

Location	Stage/Parts	Aloe-emodin	Emodin	Chrysophanol	Physcion	Total (µg/mg)
Perugia	PF-R	3.10 ± 0.30	nd	0.51 ± 0.06	15.0 ± 2.0	18.3
	PF-AP	0.82 ± 0.08	nd	1.60 ± 0.70	1.70 ± 0.90	4.12
	F-R	0.72 ± 0.07	nd	2.00 ± 1.00	0.77 ± 0.09	3.47
	F-AP	0.81 ± 0.06	nd	nd	5.00 ± 1.00	6.20
	F-Fl	2.70 ± 0.30	nd	0.69 ± 0.04	2.40 ± 0.50	5.81
Novele	PF-R	2.00 ± 1.00	nd	nd	18.0 ± 3.0	19.5
	PF-AP	0.85 ± 0.07	nd	1.51 ± 0.9	5.00 ± 1.00	7.57
	F-R	0.65 ± 0.09	nd	1.10 ± 0.50	1.00 ± 0.40	2.72
	F- AP	0.65 ± 0.08	nd	nd	3.00 ± 1.00	4.07
	F-Fl	0.71 ± 0.05	nd	0.39 ± 0.04	0.84 ± 0.08	1.94
Pescosansonesco	PF-R	1.00 ± 0.20	0.82 ± 0.09	2.00 ± 1.00	6.00 ± 1.00	10.6
	PF-AP	1.00 ± 0.30	nd	nd	4.00 ± 1.00	5.39
	F-R	1.10 ± 0.20	nd	2.00 ± 1.00	5.00 ± 1.00	5.81
	F- AP	2.00 ± 1.00	nd	0.44 ± 0.04	4.87 ± 2.00	7.41
	F-Fl	2.00 ± 1.00	nd	0.60 ± 0.07	8.00 ± 1.00	11.0

* Values expressed are means ± S.D. of three simultaneous measurements. nd: not detected, PF: preflowering plant, F: flowering plant, R: roots, AP: aerial parts, Fl: flowers. Rhein was not detected in any sample.

**Table 4:** Heavy metals (Zn, Cd, Pb and Cu) content of different parts of *A. lutea* collected from three different locations in Italy *.

Location	Stage/Parts	Zn (ppm)	Cd (ppm)	Pb (ppm)	Cu (ppm)
Perugia	PF-R	1100 ± 300	<LoD	<LoD	1100 ± 350
	PF-AP	<LoD	<LoD	<LoD	<LoD
	F-R	500 ± 100	90 ± 30	<LoD	<LoD
	F-AP	53 ± 5	<LoD	<LoD	35 ± 6
	F-Fl	300 ± 50	<LoD	<LoD	<LoD
Novele	PF-R	460 ± 90	700 ± 200	<LoD	<LoD
	PF-AP	<LoD	<LoD	<LoD	<LoD
	F-R	140 ± 40	<LoD	<LoD	<LoD
	F-AP	250 ± 20	50 ± 10	180 ± 40	<LoD
	F-Fl	190 ± 50	130 ± 30	70 ± 30	<LoD

* Values expressed are means ± S.D.; <LoD = below limit of detection; PF: preflowering plant, F: flowering plant, R: roots, AP: aerial parts, Fl: flowers. For Pescosansonesco samples (PF-R, PF-AP, F-R, F-AP, F-Fl) all values are <LoD.

**Table 5:** Antioxidant evaluation and metal chelating activity of different parts of *A. lutea* collected from three different locations in Italy *.

Location	Stage/Parts	DPPH Scavenging **	ABTS Scavenging **	FRAP **	CUPRAC **	Phosphomolybdenum Assay ^‡^	Metal Chelating Activity †
Perugia	PF-R	39.8 ± 0.5 ^a^	56.0 ± 2.0 ^a^	37.0 ± 1.0 ^b^	57.5 ± 1.5 ^a^	0.91 ± 0.05 ^a^	12.8 ± 0.7 ^a^
	PF-AP	68.0 ± 1.0 ^b^	74.0 ± 3.0 ^c^	64.0 ± 2.0 ^b^	86.0 ± 2.0 ^b^	0.87 ± 0.07 ^b^	16.0 ± 2.0 ^b^
	F-R	43.8 ± 0.1 ^a^	75.0 ± 4.0 ^a^	52.0 ± 4.0 ^a^	71.0 ± 0.9 ^a^	1.03 ± 0.07 ^a^	8.5 ± 0.1 ^a^
	F-AP	86.5 ± 1.5 ^b^	102 ± 3 ^b^	84.0 ± 2.0 ^b^	108 ± 1 ^b^	0.93 ± 0.09 ^b^	18.0 ± 2.0 ^a^
	F-Fl	52.0 ± 1.0 ^b^	63.0 ± 2.0 ^b^	50.0 ± 1.0 ^b^	65.0 ± 1.0 ^b^	0.73 ± 0.04 ^b^	15.2 ± 0.6 ^a^
Novele	PF-R	30.4 ± 0.3 ^b^	57.0 ± 4.0 ^a^	42.0 ± 2.0 ^a^	52.0 ± 2.0 ^b^	0.71 ± 0.04 ^c^	7.3 ± 0.1 ^b^
	PF-AP	58.6 ± 0.5 ^c^	121 ± 3 ^a^	65.0 ± 1.0 ^b^	82.0 ± 3.0 ^b^	0.99 ± 0.06 ^a^	19.7 ± 0.4 ^a^
	F-R	32.0 ± 1.0 ^b^	56.0 ± 2.0 ^b^	42.8 ± 0.9 ^b^	60.0 ± 2.0 ^b^	0.92 ± 0.04 ^c^	5.8 ± 0.1 ^c^
	F- AP	75.5 ± 0.3 ^c^	97.0 ± 1.0 ^c^	68.0 ± 1.0 ^c^	86.7 ± 0.6 ^c^	0.79 ± 0.01 ^c^	13.1 ± 1.5 ^c^
	F-Fl	44.0 ± 1.0 ^c^	54.0 ± 2.0 ^c^	45.5 ± 0.4 ^c^	59.0 ± 1.0 ^c^	0.77 ± 0.05 ^b^	12.9 ± 0.3 ^b^
Pescosansonesco	PF-R	20.5 ± 0.3 ^c^	36.0 ± 1.0 ^b^	29.0 ± 1.0 ^c^	40.4 ± 0.3 ^c^	0.80 ± 0.04 ^b^	6.5 ± 0.7 ^c^
	PF-AP	70.9 ± 0.4 ^a^	85.0 ± 1.0 ^b^	75.0 ± 1.0 ^a^	92.2 ± 0.6 ^a^	1.00 ± 0.10 ^a^	15.4 ± 1.5 ^b^
	F-R	26.0 ± 0.8 ^c^	53.6 ± 0.8 ^c^	38.0 ± 1.0 ^c^	46.0 ± 1.0 ^c^	0.99 ± 0.07 ^b^	6.9 ± 0.9 ^b^
	F-AP	90.4 ± 0.4 ^a^	180 ± 1 ^a^	128 ± 4 ^a^	160 ± 1 ^a^	1.43 ± 0.01 ^a^	16.3 ± 0.1 ^b^
	F-Fl	64.0 ± 1.0 ^a^	89.0 ± 1.0 ^a^	62.0 ± 1.0 ^a^	86.0 ± 1.0 ^a^	0.96 ± 0.06 ^a^	14.5 ± 0.7 ^a^

* Values expressed are means ± S.D. of three simultaneous measurements. ** mg TE/g extract (TE = Trolox equivalents); ^‡^ mmol TE/g extract; ^†^ (mg EDTAE/g extract) (EDTAE = EDTA equivalents). Data marked with different letters within the same column indicate statistically significant differences in the same stages/parts for each location (*p* < 0.05).

**Table 6:** Enzyme inhibitory effects of different parts of *A. lutea* collected from three different locations in Italy *.

Location	Stage/Parts	AChE Inhibition **	BChE Inhibition **	Tyrosinase Inhibition ^†^	α-Amylase Inhibition ^‡^	α-Glucosidase Inhibition ^‡^
Perugia	PF-R	1.74 ± 0.07 ^c^	2.03 ± 0.06 ^a^	12.0 ± 2.0 ^a^	0.39 ± 0.01 ^a^	na
	PF-AP	1.59 ± 0.01 ^c^	na	7.5 ± 0.3 ^c^	0.45 ± 0.01 ^a^	14.0 ± 0.1 ^b^
	F-R	2.20 ± 0.40 ^a^	1.90 ± 0.20 ^b^	14.0 ± 0.2 ^a^	0.45 ± 0.03 ^a^	2.1 ± 0.4 ^b^
	F-AP	1.77 ± 0.09 ^b^	0.31 ± 0.06 ^b^	7.0 ± 2.0 ^c^	0.41 ± 0.04 ^c^	na
	F-Fl	1.65 ± 0.05 ^a^	0.77 ± 0.03 ^b^	23.0 ± 2.0 ^a^	0.45 ± 0.01 ^b^	32.1 ± 0.2 ^b^
Novele	PF-R	1.88 ± 0.08 ^b^	2.02 ± 0.04 ^a^	12.0 ± 1.0 ^a^	0.39 ± 0.01 ^a^	15.8 ± 0.3 ^b^
	PF-AP	1.82 ± 0.04 ^a^	1.10 ± 0.10 ^b^	15.0 ± 2.0 ^b^	0.41 ± 0.01 ^b^	0.25 ± 0.02 ^c^
	F-R	1.92 ± 0.04 ^b^	2.05 ± 0.02 ^a^	21.0 ± 1.0 ^a^	0.39 ± 0.01 ^b^	44.2 ± 0.4 ^a^
	F-AP	1.77 ± 0.06 ^b^	1.06 ± 0.01 ^a^	21.0 ± 2.0 ^b^	0.43 ± 0.01 ^b^	2.01 ± 0.01 ^b^
	F-Fl	1.71 ± 0.04 ^a^	1.37 ± 0.07 ^a^	12.0 ± 2.0 ^c^	0.45 ± 0.01 ^b^	42.7 ± 0.3 ^a^
Pescosansonesco	PF-R	1.92 ± 0.02 ^a^	1.83 ± 0.05 ^b^	12.0 ± 1.0 ^a^	0.37 ± 0.01 ^b^	19.3 ± 0.2 ^a^
	PF-AP	1.67 ± 0.05 ^b^	1.36 ± 0.04 ^a^	20.0 ± 2.0 ^a^	0.46 ± 0.03 ^a^	37.4 ± 0.9 ^a^
	F-R	1.88 ± 0.02 ^c^	1.60 ± 0.20 ^c^	14.0 ± 2.0 ^b^	0.38 ± 0.02 ^b^	na
	F-AP	2.10 ± 0.60 ^a^	na	25.3 ± 0.5 ^a^	0.51 ± 0.01 ^a^	34.1 ± 0.4 ^a^
	F-Fl	1.51 ± 0.08 ^b^	0.58 ± 0.07 ^c^	18.8 ± 0.8 ^b^	0.48 ± 0.01 ^a^	na

* Values expressed are means ± S.D. of three simultaneous measurements. ** mg GALAE/g extract (GALAE = galantamine equivalents); ^†^ mg KAE/g extract (KAE = kojic acid equivalents); ^‡^ mmol ACAE/g extract (ACAE = acarbose equivalents); na: not active. PF: preflowering plant, F: flowering plant, R: roots, AP: aerial parts, Fl: flowers. Data marked with different letters within the same column indicate statistically significant differences in the same stages/parts for each location (*p* < 0.05).
